# Investigating the Local Effectiveness of Carbon Ion Radiotherapy for Unresectable Female Genital Tract Melanomas: A Preliminary Real-World Study

**DOI:** 10.3390/cancers16244147

**Published:** 2024-12-12

**Authors:** Amelia Barcellini, Giulia Fontana, Alessandro Vai, Giovanni Damiano Aletti, Alexandra Charalampopoulou, Silvia Chiellino, Antonino Ditto, Fabio Landoni, Laura Deborah Locati, Giorgia Mangili, Fabio Martinelli, Federica Piccolo, Jessica Franzetti, Sara Imparato, Francesco Raspagliesi, Ester Orlandi

**Affiliations:** 1 Department of Internal Medicine and Therapeutics, University of Pavia, 27100 Pavia, Italy; 2 Radiation Oncology Unit, Clinical Department, CNAO National Center for Oncological Hadrontherapy, 27100 Pavia, Italy; 3 Clinical Department, CNAO National Center for Oncological Hadrontherapy, 27100 Pavia, Italy; 4 Medical Physics Unit, Clinical Department, CNAO National Center for Oncological Hadrontherapy, 27100 Pavia, Italy; 5 Department of Gynecologic Surgery, IRCCS European Institute of Oncology, 20141 Milan, Italy; 6 Department of Oncology and Hemato-Oncology, University of Milan, 20122 Milan, Italy; 7 Hadron Academy PhD Course, University School for Advanced Studies (IUSS), 27100 Pavia, Italy; 8 Radiobiology Unit, Development and Research Department, CNAO National Center for Oncological Hadrontherapy, 27100 Pavia, Italy; 9 Department of Oncology, Fondazione IRCCS Policlinico San Matteo, 27100 Pavia, Italy; 10 Department of Gynecologic Oncology, Fondazione IRCCS Istituto Nazionale dei Tumori, 20133 Milan, Italy; 11 Department of Gynecologic Oncology, Centro di Riferimento Oncologico, National Cancer Institute, 33081 Aviano, Italy; 12 Department of Medicine and Surgery, University of Milan-Bicocca, 20126 Milan, Italy; 13 Division of Gynecologic Surgery, IRCCS Fondazione San Gerardo dei Tintori, 20900 Monza, Italy; 14 Medical Oncology Unit, Istituti Clinici Scientifici Maugeri IRCCS, 27100 Pavia, Italy; 15 Obstetrics and Gynecology Unit, IRCCS San Raffaele Scientific Institute, 20132 Milan, Italy; 16 Department of Gynecologic Oncology, Humanitas San Pio X, 20159 Milan, Italy; 17 Department of Biomedical Science, Humanitas University, 20072 Milan, Italy; 18 Radiotherapy Unit, Ospedale di Circolo Fondazione Macchi, 21100 Varese, Italy; 19 Radiology Unit, Clinical Department, CNAO National Center for Oncological Hadrontherapy, 27100 Pavia, Italy; 20 Department of Clinical, Surgical, Diagnostic, and Pediatric Sciences, University of Pavia, 27100 Pavia, Italy

**Keywords:** carbon ion radiotherapy, gynecological melanoma, local control, rare cancers

## Abstract

**Background/Objectives**: Primary gynecological melanomas are rare malignancies with lower survival rates compared to cutaneous melanomas. Both preclinical and clinical data support the evidence that mucosal melanomas are photon-radioresistant but responsive to carbon ion radiotherapy (CIRT). The aim of this study is to assess, in a real-world cohort, the effectiveness and tolerability of radical CIRT in patients with inoperable gynecological mucosal melanoma. **Methods**: This is a real-world study aimed to assess the effectiveness and the safety of CIRT in this setting. We defined as the primary endpoints the objective response rate (ORR) and the clinical benefit (CB). The secondary endpoints included the actuarial local control rate (LC) assessed after 1 year and 2 years and the toxicity scored according to CTCAE v.5. Actuarial outcomes were analyzed using the Kaplan–Meier method, while potential predictors were investigated through the Log-rank test. **Results**: Between 2017 and 2023, eleven Caucasian patients underwent pelvic CIRT (total dose 68.8 GyRBE) for mucosal malignant melanoma of the vulva or the vagina. With a median follow-up of 18 months, we observed an ORR of 82% and a CB of 100%. LC at 1 and 2 years was 100% and 86%, respectively, and among the factors analyzed for their potential impact on LC, age < 60 years seems to be a potential predictor (*p* = 0.014). The treatment was well tolerated, with only one case of acute grade 3 erythema and, in the late phase, one case of grade 3 erythema and grade 3 urethral toxicity. **Conclusions**: CIRT was effective and safe for gynecological melanomas. Larger collaborative cohort studies and longer follow-ups are needed to take a step forward in comprehending the correct management of this disease.

## Simple Summary

Gynecological mucosal melanomas are rare, aggressive, and radioresistant neoplasms for which no standardized treatment protocols currently exist. When surgery, which appears to be the gold standard, is not feasible, radiation therapy can be considered. However, mucosal melanomas exhibit specific biological hallmarks of radioresistance, and, indeed, the outcomes reported in the literature on the use of photons in this context are not encouraging. Currently, there are limited data on the use of carbon ion radiotherapy (CIRT) in this setting. The aim of this study is to evaluate, in a real-world cohort, the efficacy and tolerance of CIRT for gynecological melanomas, with the goal of expanding knowledge on the use of this radiotherapy technique in such a radioresistant and difficult-to-treat malignancy.

## 1. Introduction

Mucosal melanomas are rare entities that differ from cutaneous melanomas epidemiologically and molecularly. Indeed, despite the same embryologic origin, skin and mucosal melanocytes differ in the adhesion molecules and intracellular signalling pathways that regulate their growth and present different molecular mutations [1].

Among mucosal melanomas, those of the lower female genital tract are extremely uncommon. It is estimated that the incidence of vulvar melanomas is approximately 0.48–1.4 per 1,000,000 women per year, while vaginal melanomas account for about 0.4–0.8% of all female melanomas. Cervical melanomas are exceedingly rare, with only around 80 cases reported in the literature [1,2]. Despite therapeutic advances in the gynecological field, women with genital melanoma have not seen a substantial improvement in survival over the past few decades, particularly when the tumour is locally advanced, recurrent, or metastatic. Overall, the prognosis is scant, with a 5-year overall survival (OS) of 37–50% for vulvar, 13–32% for vaginal, and approximately 10% for cervical melanoma [1]. Currently, there are no consensus for managing gynecological mucosal melanomas, and the treatment largely relies on data from skin melanomas or histologically different gynecological cancers. Whenever feasible, radical surgery remains the gold standard, especially in the early stages. However, due to late diagnosis and the critical proximity to the lower gastrointestinal tract, urethra, and bladder, radical surgery may be challenging or, considering the consequent surgical sequelae, often declined by the patients [2,3,4,5]. When surgery is not an option, immunotherapy appears to be a promising alternative, especially when combined with radiotherapy (RT) [6].

However, mucosal melanoma cells exhibit radioresistance due to their high efficiency in repairing sublethal DNA damage following photon irradiation [7], a process which seems to be overcome by carbon ion radiotherapy (CIRT) [8]. CIRT combines physical and radiobiological advantages over photons, enabling high dosage conformations and around 3-fold higher relative biological effectiveness [RBE] in the target region. Recent radiobiological studies conducted in both 2D and 3D models have illustrated the advantages of CIRT over photons in treating vaginal melanoma. These advantages include reduced cell survival, decreased migration and invasion capacity, increased cytopathic effects, and altered melanin production (with lower production observed under CIRT) [9,10]. The significant reduction in melanin production after CIRT highlights the distinctive cellular impact of this type of RT considering the defence role of melanin after exposure to irradiation [11,12,13].

The largest clinical series regarding the use of CIRT as a curative approach in gynecological mucosal melanomas derives from a Japanese multicenter retrospective experience featuring 37 patients. These women were treated with 57.6 GyRBE- 64 GyRBE on a macroscopic tumour, achieving 2-year local control (LC), overall survival (OS), and progression-free survival (PFS) rates of 71%, 53%, and 29%, respectively [14]. Waiting for the results of the ongoing CYCLE trial, a prospective phase II study aimed at assessing the role of CIRT in gynecological melanomas [15], and in light of our previous pilot experience [16], we performed a retrospective query on our longitudinal clinical registry (NCT05203250) [17] to evaluate—in a real-world context—the local efficacy and toxicity pattern of radical CIRT delivered in women with gynecological melanomas.

## 2. Materials and Methods

This is a pilot, real-world study involving patients included in the longitudinal registry REGAL [17] who were treated with CIRT at the National Center for Oncological Hadrontherapy (CNAO) in Pavia (Italy) for unresectable gynecological mucosal melanoma. The institutional review board approved this study (CNAO OSS 68 2024-Particle GYN on 13 August 2024).

The inclusion criteria were the following: (i) age ≥ 18 years; (ii) histological diagnosis of primary or recurrent vaginal or vulvar mucosal melanoma; (iii) candidates for a curative treatment in a multidisciplinary tumour board; (iv) ineligibility for surgery and/or patient’s refusal; (v) CIRT delivered as a radical approach with a prescription dose to the gross tumour volume (GTV) of 68.8 GyRBE; (vi) performance status = 0–1 according to Eastern Cooperative Oncology Group (ECOG) score; and (vii) possibility to perform magnetic resonance imaging (MRI). The combination with other oncological systemic therapy, when indicated, was allowed. We excluded (i) patients enrolled in the ongoing phase II prospective study (NCT05478876) [15], (ii) patients treated with a not-radical intent, and (iii) a prescription dose to GTV < 68.8GyRBE.

The co-primary endpoints, defined on a “per lesion” basis, were the following:

(i) the objective response rate (ORR), which is the maximum reaction after CIRT, as evaluated through MRI and gynecological clinical assessments. For the aim of this study, we considered ORR as the sum of complete response (CR) and partial response (PR).
(ii) the clinical benefit (CB) as a composite of objective response and stable disease (SD).

The secondary endpoints included the following: (i) the 1-year (1-y) and 2-year (2-y) actuarial LC rates, where the LC was the time interval between the start of CIRT and the evidence of a progression of disease (PD) in the field of treatment; and (ii) the toxicity rates evaluated during the treatment, as well as the follow-up according to the CTCAE version 5.0 scales [18]. Concerning toxicity, we considered acute toxicities occurring within six months after CIRT and late toxicities occurring beyond six months.

### 2.1. CIRT Procedures and Follow-Up

Tailor-made fixation cushions and thermoplastic shells were built and used as patient immobilization systems in the planning computed tomography (CT), which was acquired in the supine position. The GTV was defined based on a planning CT rigidly registered with contrast-enhanced MRI for all lesions. The definition of treatment volumes, the fractionation scheme, and the total dose were based on the Japanese experience described by Karasawa et al. [19] and Murata et al. [14]. The dose conversion described by Fossati et al. [20] was applied. In particular, RBE-weighted dose distributions were optimized in the treatment planning system (TPS) using the LEM-I model, the only CE-marked biological model available at the time of this study. For the plan setup, two contralateral horizontal fields were used. Indeed, considering the patient in the supine position, a vertical field would have intersected the lower urinary tract (bladder and urethra) and the intestinal tract (anus, rectum, and sigmoid colon). These organs are indeed prone to intra- and inter-fractional non-rigid anatomical variations, which could lead to reproducibility issues and dose distribution variations. 

The CIRT consisted of a total of two treatment phases using a sequential boost approach. Specifically, in line with the findings from the Japanese literature [14,19], two treatment volumes were identified. The first volume, termed clinical target volume- low dose (CTV-LD), encompassed areas with macroscopic disease, potential microscopic disease, and the lymphatic drainage regions, which received a total of 43 GyRBE (4.3 GyRBE per fraction, 10 fractions). The second volume, which included the GTV and a minimum margin of 5 mm, was referred to as the CTV high dose (CTV-HD), receiving an additional 6 fractions at the same dose per fraction, ultimately reaching a total of 68.8 GyRBE.

In the optimization process, high priority was given to adhering to constraints for organs at high risk (OARs) of toxicity, according to our clinical practice: (i) for rectum, dose at 1cc (D1cc) ≤ 66 GyRBE, D5cc ≤ 61 GyRBE, D10cc ≤ 54 GyRBE [21]; (ii) for sigmoid, the maximum dose (Dmax) ≤ 52 GyRBE; and (iii) for small and large bowel, Dmax ≤ 43 GyRBE. Plans were optimized with a TPS-embedded robust optimization tool, mitigating the impact on CTV’s coverage and OAR’s constraints of expected range uncertainties (±3.5%) and rigid setup variations (±4 mm). These parameters were selected according to shared studies and our long-term experience [22]. Moreover, plan optimization was carried out iteratively to ensure uniform biological coverage of the target while minimizing exposure to OARs. The physical dose was therefore adjusted so that, through the LEM I model, the resulting dose distribution would meet these optimization criteria.

In addition, specific configurations were implemented to address inter- and intra-fraction variations. Notably, (i) to ensure anatomical reproducibility and minimize pelvic organ movements, patients received rectal enemas and 100 mL of normal saline into the bladder during both the planning CT and each treatment fraction. Additionally, patients were advised to take laxatives to prevent constipation throughout the treatment period. (ii) Daily Cone Beam CT (CBCT) scans were performed as setup imaging to assess bladder and rectal preparation and the reproducibility of the CTVs, while (iii) weekly re-evaluative CT scans were used to monitor plan quality throughout treatment delivery. Decisions regarding plan re-optimization and treatment adjustments were based on daily CBCT and/or weekly CT assessments [23,24].

Clinical evaluations were conducted at least once a week during treatment. Following CIRT, patients were re-evaluated every 3–4 months both radiologically (MRI) and clinically by a radiation oncologist and/or a gynecological oncologist, performing a gynecological clinical assessment to evaluate the local control. CT and/or PET/CT were routinely performed as restaging along with additional radiological examinations, if there was suspicion of disease progression.

### 2.2. Statistical Analysis

Continuous variables were presented as medians with interquartile (IQR) ranges, while categorical variables were described using counts and percentages. Actuarial outcomes were assessed using the Kaplan–Meier method, and the Log-rank test was used to explore any potential predictors. Quantitative variables were stratified based on their median value, and cut-offs in the literature were explored. The 1y-LC, 2y-LC, ORR, and CB rates were provided with their binomial 95% confidence intervals (95% CI), and the two-sided type I error was set to 0.05. Statistical analyses were carried out using R version 4.0.1.

## 3. Results

### 3.1. Patient, Tumour, and Treatment Characteristics

Between 2017 and 2023, eleven Caucasian patients not included in the ongoing phase II prospective clinical trial NCT05478876 [15] underwent pelvic CIRT for mucosal malignant melanoma of the lower female genital tract. The median age at CIRT was 72 years (range: 52–87 years). Seven (64%) patients had vaginal cancer and four (36%) had vulval tumours. In four cases (36%), patients were enrolled for CIRT for post-surgical recurrence. One patient received CIRT for re-irradiation. All patients were BRAF, KRAS, and NRAS wild type, but three cases (27%) carried the c-kit mutation. The GTV ranged between 13.2 and 133.07 cm^3^. The median follow-up period was 18 months (range: 4–34 months) for all patients. No concomitant systemic therapies were administered during CIRT; immunotherapy was stopped during CIRT, and, overall, seven patients received (five before and also after, while two only after) immune checkpoint inhibitors (ICIs). The patient, tumour, and treatment characteristics are shown in Table 1 and Table 2.

### 3.2. Outcomes According to the Endpoints

**Figure 1:**
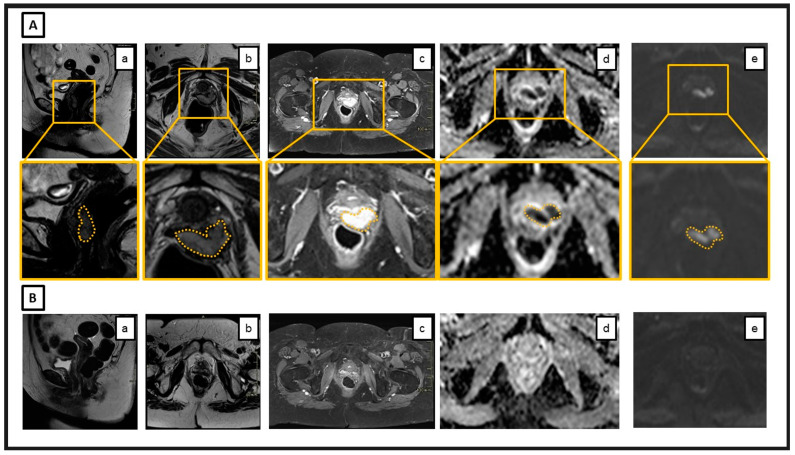
The panel (**A**) shows baseline magnetic resonance images (MRI): a lesion originating from the left anterior vaginal wall can be observed, involving the middle–lower third of the vaginal canal and extending beyond the paracolpium in the left anterolateral area. This lesion shows an intermediate signal on T2 (**a**,**b**), with homogeneous and regular contrast enhancement (**c**), and marked signal restriction on diffusion sequences (**d**,**e**). For each sequence, an enlargement of the disease (outlined in ochre) is provided. In panel (**B**), the MRI images correspond to the post-CIRT follow-up and document a complete response to the treatment. Specifically, the previously described tissue in the vagina is no longer recognizable (**a**,**b**). There are no areas of pathological contrast enhancement (**c**) nor signal restriction on the diffusion sequences (**d**,**e**).

**Figure 2:**
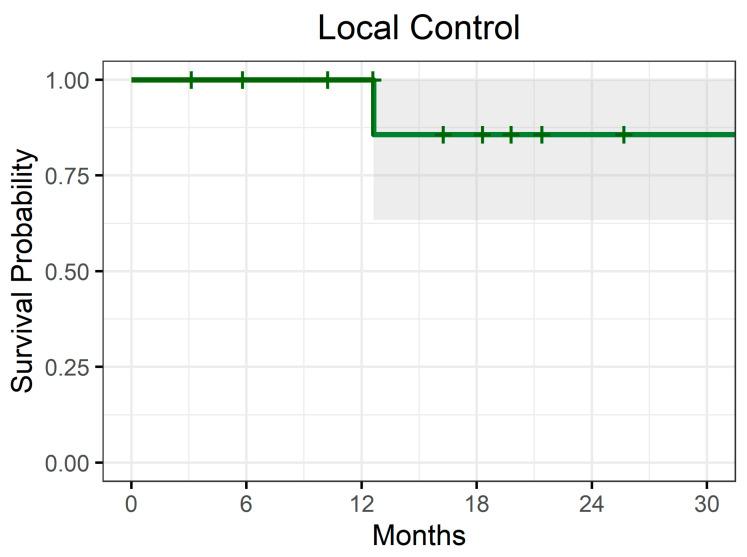
Kaplan–Meier survival probability curve for local control of the entire cohort.

Within 6 months after CIRT, three lesions (27.3%, 95% CI: 0.95–53.6%) achieved CR (Figure 1), while six (54.5%, 95% CI: 25.1–84.0%) a PR within 13 months, and two (18.2%, 95% CI: 0–41.0%) an SD within 10 months. Overall, the ORR was 82% with a CB of 100%. The CB was maintained for a median time of 16 months (range 3–33 months). We observed that the administration of ICIs before and/or immediately (<1 month) after CIRT (yes/no), GTV (>/≤28 cm^3^), and age (>/≤72 years) were unrelated to the achievement of ORR (Table 3). After a median follow-up of 18 months (IQR: 8.7, 20.3), two patients (18%) experienced a loco-regional relapse, and, overall, the 1-y and 2-y LC were 100% and 86%, respectively (Figure 2). Patients with an age >60 years seemed to experience a better LC (*p* = 0.014). The administration of ICIs before and/or immediately (<1 month) after CIRT (yes/no, *p* = 0.68), GTV (>/≤28 cm ^3^, *p* = 0.25), the tumour localization (vagina vs. vulva, *p* = 0.39), CIRT at first diagnosis or at recurrence (*p* = 0.39), the mutational status (wild type vs. c-kit mutation, *p* = 0.53), and the achievement of ORR (yes/no, *p* = 1) were not significantly related to the higher chance to achieve an LC.

Regarding toxicity, the treatment showed a high safety profile; indeed, CIRT was well tolerated, and no interruption was needed. Except for one case of grade 3 erythema, no major intra-treatment toxicities were recorded. Among the major toxicities, defined as grade ≥ 3 during follow-up, there was one case of grade 3 erythema and one case of grade 3 urethral stricture (Table 4). We also observed two cases of vitiligo after the resumption of ICI post-CIRT in patients who underwent sandwich immunotherapy (with a suspension of up to 15 days before and after CIRT, according to our clinical practice).

## 4. Discussion

Gynecological mucosal melanomas, often described as “rare among the rare,” present diagnostic and therapeutic challenges. With only an estimated two cases diagnosed annually in specialized centres, the lack of consensus and guidelines makes effective management challenging, even for highly skilled physicians [5,25]. Despite advancements in treatment and the implementation of immunotherapy in this setting, patient outcomes continue to be suboptimal and variably predictable. The rarity of the disease prevents the planning of randomized studies, and our current knowledge relies on small experiences, case series, and case reports, most of which are retrospective, following an approach based on experience gained in treating skin melanomas or other gynecological tumour histologies. This limits the possibility of reaching high levels of evidence regarding the optimal therapeutic options in this challenging setting. The most frequently adopted approach is surgery; however, in advanced or recurrent cases, it may be not feasible or lead to comorbidities that delay the subsequent adjuvant treatment [1]. Nevertheless, when the tumour multidisciplinary board considers a local approach but surgery is excluded, clinicians should keep in mind the suboptimal effectiveness of a traditional photon-based RT. Indeed, melanoma cells exhibit several radioresistant hallmarks: effective enzymatic system, rapid proliferation and strong repair capability, high degree of poor differentiated and hypoxic cells (including the cancer stem cells) and abnormal apoptosis [26], as well as intrinsic melanogenesis process (which seems to increase the resistance to ionizing radiation) [9]. However, it has been demonstrated that CIRT is able to overcome these characteristics in a dose-dependent manner, affecting the survival rates of melanoma cells [10,27,28]. From a clinical perspective, the local effectiveness of CIRT has been well documented, particularly in series of head and neck melanomas [29,30,31,32,33,34]. At present, a key barrier to the implementation of CIRT on a larger scale has been unequal access around the world. To date, only 13 ion beam facilities worldwide offer CIRT for both preclinical and clinical applications across Asia and Europe [35].

Although the small sample size and the short follow-up limit the statistical strength of the conclusions, our real-world study suggests the clinical effectiveness of CIRT in gynecological melanomas. We experienced 82% of ORR and 100% of CB in a series of patients homogeneous, from a RT perspective, in terms of the total dose, planning, technique, and radiobiological model used. This makes our data encouraging, especially when compared to photon studies, which consist of case reports, where RT protocols, total doses, and techniques are inconsistent and the time span is extensive, resulting in heterogeneous data in terms of the technique and technology used [36]. The consistency of the RT protocol, which is a strength of our analysis, allows for a meaningful comparison with the largest multicentre series of gynecological melanomas treated with radical RT. We are referring to the multicentre retrospective analysis carried out by the Japanese gynaecologic working group featuring 37 patients (of whom 22 had vaginal, 12 vulvar, and 3 cervical uterine melanomas) treated with CIRT across 13 years. Consistently with our series, the Japanese experiences reported 100% CB, considering that, within 6 months, 19 patients experienced CR, 14 PR and 4 SD. The ORR at 6 months would correspond to 89.2%; however, among the 18 women who did not experience a CR within 6 months, 11 obtained it in a late phase [14]. The slight difference in ORR between our study and the Japanese cohort might be attributable to our limited sample size and the different radiobiological models used in plan optimization [37].

Our LC data are encouraging when compared to surgical data, where vulvovaginal melanomas recur in up to 42–70% [1,2,38,39,40], making CIRT a valuable local option to consider. We reached 1- and 2-y LC rates of 100% and 86%, confirming the high rates reported by Murata et al. [14] (2- and 5-year rates of 71% and 44%). While the risk of local recurrence seems to be related to the tumour size in the surgical literature [1,2,41], a large tumour volume did not significantly influence the LC after CIRT. These data, along with the mild toxicity profile observed in this setting, support CIRT as a radical approach in locally advanced gynecological melanomas. Similarly to the Japanese series, the toxicities recorded in our cohort were medically manageable and not higher than grade 3. These mild toxicities appeared to be less impactful than the usual post-surgical sequelae, especially in cases where alternative radical treatment is a pelvic exenteration. It is interesting to note that, in line with the recent literature [30,42], the prior or subsequent administration of ICIs does not increase the frequency of any adverse events of grade ≥3, indicating the favourable safety profile of the combination approach. Intriguing are the two cases of vitiligo which arose after CIRT and during the resumption of ICIs. The data in the literature reported a stronger pro-immunogenic effect of CIRT compared to photon beam RT. On one hand, the spatial selectivity of CIRT leads to a smaller amount of chromosomal abnormalities in peripheral blood cells, increasing the possibility of triggering a stronger immune response [43,44]. On the other, CIRT increases the release of immune-stimulating cytokines [43], causing a stronger immune response in the tumour microenvironment [45,46]. Considering this pro-immunogenic effect, the ICI’s mechanism, and the toxicity which occurred after CIRT only in patients who underwent a “CIRT-ICIs sandwich approach”, it is plausible for CIRT to have contributed to triggering this adverse event in a tissue (the skin) that is physiologically characterized by a high level of immune infiltration.

Waiting for the results of the CYCLE trial which will assess, prospectively, the role of CIRT in selected gynecological melanomas [15], to the best of our knowledge, this is the largest series of Caucasian patients treated with CIRT for female genital tract melanoma. As previously specified, the small number of enrolled patients is an undeniable limitation and prevents us from drawing definitive conclusions, but it is consistent with the incidence of this disease and the enrolment years. Due to the rarity of these malignancies, we strongly believe that the only feasible way to expand our knowledge, enhance treatment, and improve prognosis is by pooling real-world data from expert centres. This requires an intense and collaborative national and international effort, and the REGAL registry [17] and MITO-9-vulva [47] might be among one of the first steps. Otherwise, there is a real risk of underrepresenting gynecological melanomas within clinical trials because they are too rare to reach a reasonable sample size or losing them within large sample sizes that combine heterogeneous conditions [48].

## 5. Conclusions

Herein, we described the high local effectiveness of CIRT, accompanied by a high safety profile, in a real-world series of gynecological mucosal melanomas. Considering the incidence of this disease, a strong international collaboration among experts in rare gynecological malignancies is warranted to prevent gynecological mucosal melanomas, and their treatment with CIRT, from being overlooked even when included in clinical research.

## Figures and Tables

**Table 1:** Patient, tumour, and treatment characteristics.

Characteristics	Median (IQR)
Age	72.0 years (62.0, 81.5)
GTV	28.0 cm^3^ (25.5, 43.7)
	Number (%)
Histology	
Epithelioid	1 (9.1%)
Epithelioid and spindle-shaped cells	1 (9.1%)
Melanoma NOS	7 (64%)
Nodular	1 (9.1%)
Small round cells	1 (9.1%)
Recurrence after surgery	4 (36%)
First diagnosis	7(64%)
Mutational status	
BRAF wild type	11(100%)
NRAS wild type	11(100%)
KRAS wild type	11(100%)
c-kit mutated	3 (27%)
Primitive site	
Vagina	7 (64%)
Vulva	4 (36%)

**Table 2:** Clinical Tumor-Node-Metastasis (TNM) staging at the time of CIRT according to the AJCC (American Joint Commission on Cancer)-8th Edition, suggested for the melanoma staging. For each case, the corresponding FIGO (International Federation of Gynecology and Obstetrics) classification is also reported [1].

Patient	Localization	TNM (AJCC)	FIGO
P1	vagina	Tx N0 M0	II
P2	vagina	T4a N0 M0	II
P3	vagina	Tx N0 M1a	IVB
P4	vagina	Tx N2 M0	III
P5	vagina	T4b N0 M0	II
P6	vagina	T4b N0 M0	II
P7	vagina	Tx N0 M0	II
P8	vulva	T4b N0 M1a	IVB
P9	vulva	T4a N2 M0	IIIB
P10	vulva	T2a N0 M0	IIIA
P11	vulva	T4b N0 M1a	IVB

**Table 3:** Impact of analyzed variables on the objective response rate.

		Objective Response		
Variable	Overall, N = 11 ^1^	No, N = 2 ^1^	Yes, N = 9 ^1^	*p*-Value ^2^
**Age [years]**	72.0 (62.0, 81.5)	70.5 (64.8, 76.3)	72.0 (62.0, 81.0)	0.906
**Age > 72 [years]**	5 (45%)	1 (50%)	4 (44%)	>0.999
**Age > 60 [years]**	9 (82%)	1 (50%)	8 (89%)	0.345
**Histology**				>0.999
Epithelioid	1 (9.1%)	0 (0%)	1 (11%)	
Epithelioid and spindle-shaped cells	1 (9.1%)	0 (0%)	1 (11%)	
Melanoma NOS	7 (64%)	2 (100%)	5 (56%)	
Nodular	1 (9.1%)	0 (0%)	1 (11%)	
Small round cells	1 (9.1%)	0 (0%)	1 (11%)	
**Mutational Status**				0.491
c-kit mutated	3 (27%)	1 (50%)	2 (22%)	
c-kit wild type	8 (73%)	1 (50%)	7 (78%)	
**Primitive Site**				0.491
Vagina	7 (64%)	2 (100%)	5 (56%)	
Vulva	4 (36%)	0 (0%)	4 (44%)	
**First Diagnosis Treatment**	7 (64%)	2 (100%)	5 (56%)	0.491
**Immunotherapy**	7 (64%)	0 (0%)	7 (78%)	0.109
**GTV [cc]**	28.0 (25.5, 43.7)	79.3 (52.5, 106.2)	28.0 (25.5, 41.3)	0.582
**GTV > 28.01 [cc]**	5 (45%)	1 (50%)	4 (44%)	>0.999
**Best Response**				0.018
Complete Response	3 (27%)	0 (0%)	3 (33%)	
Partial Response	6 (55%)	0 (0%)	6 (67%)	
Stable Disease	2 (18%)	2 (100%)	0 (0%)	
**Best Response Time [months]**	6.6 (4.2, 8.9)	6.4 (4.2, 8.5)	6.6 (4.9, 8.2)	0.909
**Local Relapse at Last Follow-Up**	2 (18%)	0 (0%)	2 (22%)	>0.999
**Follow-Up Time [months]**	18.3 (8.7, 20.3)	7.4 (5.2, 9.5)	19.8 (16.3, 20.8)	0.145

^1^ Median (IQR); n (%). ^2^ Wilcoxon’s rank-sum test; Fisher’s exact test; and Wilcoxon’s rank-sum exact test.

**Table 4:** Carbon Ion Radiotherapy Toxicity.

Toxicity	Acute Toxicity N (%)	Late Toxicity N (%)
**Skin**		
G1	-	2 (18%)
G2	3 (27%)	-
G3	-	1 (9%)
**Vagina**		
G1	6 (55%)	2 (18%)
G2	1 (9%)	
**Bone**		
G1	-	1 (9%)
**Bladder**		
G2	2 (18%)	-
**Urethra**		
G3	-	1 (9%)
**Bone**		
G1	-	1 (9%)

## Data Availability

Data will be shared upon reasonable request to the corresponding author.
